# Effects on Weight Gain and Length of Hospital Stay of Day and Night-Cycled Light Exposure in Premature Infants: A Meta-Analysis

**DOI:** 10.1097/jnr.0000000000000700

**Published:** 2025-09-04

**Authors:** Ciao-Lin Ho

**Affiliations:** Department of Child Care and Education, Hungkuang University, Taichung City, Taiwan, ROC

**Keywords:** cycled light, weight gain, length of stay, premature infants

## Abstract

**Background:**

Continuous bright light (CBL) and dim light (DL) exposure in premature babies may adversely affect their physiological and vision development and interfere with parental sleep and quality of life.

**Purpose:**

This systematic review and meta-analysis were conducted to investigate the effects of day/night cycled light (CL) exposure on weight gain and length of hospital stay (LOS) in premature infants.

**Methods:**

MEDLINE, PsycINFO, PubMed, CINAHL, Cochrane, Airiti Library, and Google Scholar were searched using appropriate keywords for relevant English or Chinese articles published before April 7, 2023. Study quality was evaluated using RevMan software (version 5.4) and the Cochrane risk-of-bias assessment tool (version 2.0). The cohort was divided into two groups based on the light exposure cycle studied, namely the intervention (CL) and control (CBL and DL) groups.

**Results:**

Ten studies were included in the meta-analysis, with the results revealing LOS was, respectively, 8.11 days (95% confidence interval [CI]=[−14.76, −1.46]; *p*=.02) and 7.21 days (95% CI=[−13.82, −0.60]; *p*=.03) less in the CL group than in the DL and CBL groups. Thus, the CL group recorded a total reduction of 7.52 days in LOS (95% CI=[−12.87, −2.17]; *p*=.006). Also, the standardized mean difference in weight gain between the CL and DL/CBL groups was 0.62 (95% CI=[0.00, 1.24]; *p*=.05).

**Conclusion/Implications for Practice:**

The findings indicate that premature infants exposed to CL experience substantially less LOS and more weight gain than their peers exposed to either DL or CBL. As premature infants require CL exposure after discharge, its implementation in the neonatal intensive care unit should be transitional and timed appropriately. Notably, the studies included in this meta-analysis used small sample sizes and were affected by a number of quality concerns. Thus, large-scale studies should be conducted to confirm the optimal duration of CL exposure for premature infants in neonatal intensive care units.

## Introduction

The average rates of preterm birth in low-income, medium-income, and high-income countries are approximately 12.0%, 9.4%, and 9.3%, respectively (Walani, 2020). Approximately 23.5% of all infants aged 5 months are unable to sleep continuously for >6 hours (Touchette et al., 2005), which has been shown to negatively affect their health as well as cause parental distress (Perrella et al., 2022), fatigue (Yan et al., 2022), depression (Hall et al., 2022; McEvoy et al., 2019), sleep disorders, stress, and reduced sense of well-being (Lee & Kimble, 2009). Improper light exposure alters the circadian rhythm of premature infants, with negative impacts on their sleep, activity, central nervous system development, and long-term health (Trickett et al., 2022). Compared with term infants, premature infants are highly susceptible to malnutrition, which is commonly associated with poor growth. Therefore, for premature infants, appropriate care measures must be adopted to promote growth, shorten the length of hospital stay (LOS), and reduce medical costs.

Premature infants are born before their organs are fully developed. Hence, the environment in the neonatal intensive care unit (NICU) must be optimal to sustain or improve their health. Lighting used in the NICU includes dim light (DL), continuous bright light (CBL), and day/night cycled light (CL). DL exposure may be defined as 24-hour exposure to irregular DL (Guyer et al., 2012), a near-darkness condition (Brandon et al., 2002), or continuous DL (Boo et al., 2002). DL may have harmful effects on the retina, which consumes higher levels of oxygen under dark conditions than bright conditions (Aquah et al., 2021). Newborns exposed to DL experience reduced heart and respiratory rates and lower systolic and diastolic blood pressure on the first and second days after birth (Abdel Hamid et al., 2021). CBL exposure is defined as 24-hour exposure to the traditional continuous light or normal room light conditions (Sánchez-Sánchez et al., 2022; Vásquez-Ruiz et al., 2014). Zores et al. (2015) reported that Lux values higher than 50 may lead to increased heart and respiratory rates as well as regional cerebral oxygen saturation in premature infants. CL exposure is defined as 12-hr exposure to light during the daytime and 12-hr exposure to darkness at night (Boo et al., 2002; Brandon et al., 2002; Farahani et al., 2018; Guyer et al., 2012; Sánchez-Sánchez et al., 2022; Seiberth et al., 1994; Vásquez-Ruiz et al., 2014). The fetus develops in the mother’s body in a natural DL environment with optimized circadian rhythm conditions, and enters a CL environment after hospital discharge. The NICU environment should be a transition period between these two light modes. Improper light exposure can affect circadian rhythm in premature infants, interfering with their sleep, activity, central nervous system development, and long-term health (Trickett et al., 2022).

A growing number of randomized and empirical studies have explored the benefits of perinatal light exposure. Evidence suggests that, compared to DL or CBL, CL is associated with better weight gain (Farahani et al., 2018), reduced LOS (Esmaeilizadeh et al., 2016; Guyer et al., 2012; Vásquez-Ruiz et al., 2014; Sánchez-Sánchez et al., 2022), optimized melatonin rhythm (Vásquez-Ruiz et al., 2014), and longer sleep–wake cycles and nighttime sleep times in premature infants (Zores-Koenig et al., 2020). Good circadian rhythms and sleep–wake cycles promote growth and weight gain in infants, facilitating timely hospital discharge. However, the findings of several studies indicate CL exerts no significant effect on weight gain or LOS (Brandon et al., 2002; Brandon et al., 2017; Seiberth et al., 1994).

The American Academy of Pediatrics has issued CL exposure guidelines for premature infants (Rodríguez et al., 2016). The slower rates of growth and lower weight gains often exhibited by premature infants in the NICU provide a sensitive indicator of malnutrition (Goldberg et al., 2018). LOS and weight gain are key factors widely recognized as influencing the risk of hospitalization in premature infants (Pereira-da-Silva et al., 2019; Rodovanski et al., 2022). In light of the above, this study was conducted to investigate the effects, specifically in comparison with DL and CBL, of CL exposure on weight gain and LOS in premature infants.

## Methods

### Guideline Adherence

The protocol for this systematic review and meta-analysis was registered with PROSPERO (protocol number: CRD42024511708). This study was conducted in accordance with Preferred Reporting Items for Systematic Reviews and Meta-Analyses guidelines (Page et al., 2021).

### Literature Search and Selection

In this study, the Population, Intervention, Comparison, and Outcome (PICO) framework was employed to investigate the effects of CL exposure on weight gain and LOS systematically in premature infants, and relevant keywords were used to identify relevant randomized controlled trials. In the context of this study, the PICO components were identified as follows: “P” indicates preterm and premature infants; “I” indicates CL, cycled lighting, a light/dark cycle, light exposure, and the circadian rhythm; “C” indicates DL and CBL; and “O” indicates body weight gain and LOS. The keyword search terms formulated using the PICO framework were “preterm infant,” “premature infant,” “cycled light,” “cycled lighting,” “a light/dark cycle,” “light exposure,” and “circadian rhythms.” The Boolean operators “OR” and “AND” were used, with “OR” used to combine synonyms. Airiti Library, MEDLINE, PsycINFO, PubMed, CINAHL, and Cochrane were searched for all relevant articles published in either Chinese or English before April 7, 2023. A literature search using Google Scholar was also conducted.

The inclusion criteria were studies conducted on premature infants that adopted CL as the intervention arm, employed either CBL or DL as the control arm, reported outcomes related to premature infant developmental indicators (e.g., weight gain, LOS), used a randomized controlled trial as the study design, and were published in either Chinese or English. The exclusion criteria were studies with irrelevant titles or abstracts and reporting outcomes unrelated to weight gain or LOS.

Two reviewers searched the literature independently based on the inclusion criteria, initially screened studies that met the inclusion criteria, and read the full texts of the selected articles. Data extracted from the selected articles included first author’s name, publication year, country, postmenstrual age (PMA), gestational age, birth weight, intervention methods, and outcomes (i.e., LOS and weight gain). Data on LOS and weight gain were collected in the CL, CBL, and DL groups. The cutoff point for measuring weight gain was set as the time after birth when the intervention was performed. Two reviewers cross-checked the extracted data, and any discrepancies were resolved through mutual discussion.

### Quality Assessment

The methodological quality of the included studies was assessed using the Cochrane risk-of-bias tool (version 2.0). The assessment focused on biases attributable to the randomization protocol, deviations from intended interventions, missing outcome data, outcome measurements, and results selection (Higgins et al., 2019). Each bias domain used signaling questions to facilitate judgments about the degree of bias risk. The response options for the signaling questions were as follows: yes, probably yes, probably no, no, and no information. Risk of bias was rated as low, some concerns, or high. Finally, two reviewers assessed the risk of bias in each domain and assigned each study a rating of “low risk,” “high risk,” or “some concerns.” Reports with >10% missing data were automatically assigned to the “high risk” of bias category.

### Statistical Analysis

The meta-analysis was performed using RevMan software version 5.4 (Biostat, Englewood, NJ; provided by the Cochrane Collaboration). In this study, effect sizes are presented as either mean difference (MD) or standardized mean difference (SMD) values with 95% confidence intervals (CIs). A random-effects model was used to estimate the treatment effects. Heterogeneity was assessed using Cochran’s Q with a *p* value of <.1, reflecting substantial heterogeneity (Higgins et al., 2019). The *I*² statistic was used to examine the degree of heterogeneity, ranging from 0 to 100, with higher scores reflecting greater between-study heterogeneity. Due to the limited number of included studies, a funnel plot could not be performed (Higgins et al., 2019). Instead, an Egger’s intercept test (Egger et al., 1997) was conducted to assess publication bias, with a significance level (*p*<.05) used to identify significant publication bias.

LOS (days) and weight gain (g/day or g/week) were evaluated as continuous variables and presented in terms of MD values. Given the variance in the units of time used to calculate weight gain across the included studies, the SMD value was calculated and used for comparison. For the one study that reported median and interquartile range values for daily weight gain in the CL and DL groups (Boo et al., 2002), the formula of (Hozo et al. 2005) was used to convert median and interquartile range values into mean and *SD* values, with the median used as the measure of central tendency for sample sizes greater than 25, and range/4 was used to estimate the *SD* (variance) for sample sizes between 15 and 70. Also, for the one study (Vásquez-Ruiz et al., 2014) that presented LOS in terms of mean±*SEM* (standard error), the mean±*SEM* values were converted into mean±*SD* values using the formula: *SD*=*SEM* × √n (sample size).

## Results

### Study Selection and Inclusion

**Figure 1:**
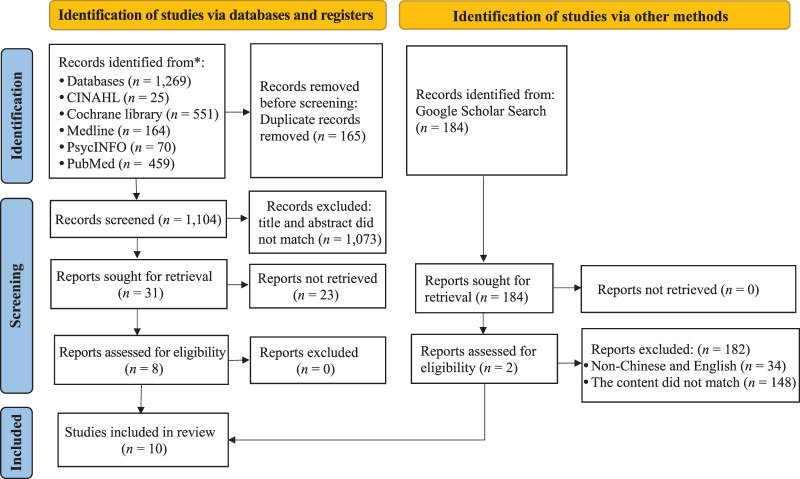
Flowchart of the Literature Search and Study Selection

A flowchart depicting study selection is presented in Figure 1. An initial search of five electronic databases returned 1,269 articles, of which 165 were duplicates. After title and abstract screening, 1,073 were found to be ineligible for reasons including: not being a randomized controlled trial, not reporting results on weight gain and LOS, and abstracts written in a language other than Chinese or English. Also, the full text was unavailable for 23 articles, resulting in their exclusion. The Google Scholar search returned two additional articles. At the end of the selection process, 10 studies (Boo et al., 2002; Brandon et al., 2002; Brandon et al., 2017; Esmaeilizadeh et al., 2016; Farahani et al., 2018; Guyer et al., 2012; Miller et al., 1995; Sánchez-Sánchez et al., 2022; Seiberth et al., 1994; Vásquez-Ruiz et al., 2014) were deemed valid and included in this review. Based on light exposure, the cohort was divided into intervention (CL) and control (DL and CBL) groups, which respectively comprised 435 and 499 premature infants.

### Characteristics of the Included Studies

The inclusion criteria and the interventions and outcomes for the two groups are shown in Table 1. The results of the meta-analysis performed to compare LOS and weight gain among the light exposure groups are shown in Figure 3. The timing of CL exposure may affect subsequent LOS and weight gain in premature infants. In the NICU, the duration of DL was higher for infants with late CL exposure than for those with early CL exposure. Hence, infants with late CL exposure may be classified as being closer to the DL group. One of the included studies compared three groups organized based on their timing of CL exposure (Brandon et al., 2002). Premature infants exposed to CL from 36 weeks PMA (the DL group) were compared with those exposed to CL from birth (the CL group 1; Brandon et al., 2002) and those exposed to CL from 32 weeks PMA (the CL group 2; Brandon et al., 2002). In another study from the same group (Brandon et al., 2017), premature infants exposed to CL at 36 weeks PMA were defined as the DL group and compared with those exposed to CL at 28 weeks PMA (the CL group).

**Table 1:** Study Characteristics

Author (Year)/Country	Inclusion Criteria PMA	Gestational Age	Birth Weight	Intervention	Outcome
1. Boo et al. (2002) / Malaysia	<37	CL: 31.6 (2.2) weeks; DL: 31.4 (2.2) weeks	CL:1,482 (236) g; DL: 1,465 (280) g	CL: 78.4 (24.7) lux during 07:00-19:00; 5.9 (1.9) lux during 19:00-07:00 DL: 5.9(1.9) lux throughout the day	CL group *n* = 47, male 31 (62.0%); DL group *n* = 44, male 22 (47.8%). Weight gain after birth: 11.9 (26.5)g/day in the CL group; DL group 12.2 (8.5)g/day
2. Brandon et al. (2002) / USA	<31	27.1 (2.0) weeks across the 3 groups	1,000 (223) g across the 3 groups	CL: 200-225 lux during 06:30-7:30;5-10 lux during 19:30-06:30 DL: 5-10 lux throughout the day, except during 6:30-7:30 and 18:30-19:30	CL group 1: CL from birth group *n* = 22; CL group 2: CL at 32 weeks’ PMA group *n*=19. DL group : CL at 36 weeks’ PMA group (considered as DL) *n* = 21; approximately half were female. LOS: CL group 1: 78 (43) days; CL group 2: 74 (41) days; DL group: 40 (21) days. Postnatal weight gain: CL group 1: 117 (138) g; CL group 2: 122 (149) g; DL group: 93 (112) g.
3. Brandon et al. (2017) / USA	<28	CL at 28 weeks’ PMA group: 26.3 (1.4) weeks; CL at 36 weeks’ PMA group: 26.3 (1.5) weeks	CL at 28 weeks’ PMA group: 874.1 (219.7) g; CL at 36 weeks’ PMA group: 872.7 (232.7) g	CL: CL at 28 weeks’ PMA group, 200-600 lux during 07:30-18:30; 5-30 lux during 18:30-07:30 DL: CL at 36 weeks’ PMA group, 5-30 lux throughout the day	CL group *n* = 61, male 39 (63.9%); DL group *n* = 57, male 26 (45.60%). LOS: CL group 91.4 (45) days; DL group 96.9 (48.2) days
4. Guyer et al. (2012) / Switzerland	<32	CL: 30.6 (0.95) weeks; DL: 29.5 (2.1) weeks	CL: 1,439 (299) g; DL: 1,284 (346) g	CL: 499.3 (159.2) lux during 07:00-19:00; 28.5 (27.5) lux during 19:00-07:00 DL: 97.6 (45.3) lux during the day; 20.8 (20.7) lux at night	CL group *n* = 17, male 8 (47%); DL group *n* = 20, male 12 (60%). LOS: CL group 41.6 (10.9) days; DL group 54.4 (23.0) days Postnatal weight gain: 30.3 (7)g/day in the CL group; 26.7 (4.7)g/day in the DL group
5. Seiberth et al. (1994) / Germany	<32	CL: 29.0 (1.7) weeks; DL: 29.3 (2.1) weeks	CL: 1,091 (233) g; DL: 1,125 (232) g	CL: NICU 342 (55) lux during the day; 62 (53) lux at night. In the premature infant ward, 415 (42) lux during the day, 26 (18) lux at night DL: Black opaque plastic patches covered with cotton cloth are placed on both eyes to reduce gloss by more than 99.9%	CL group *n* = 65, male 23 (35.4%); DL group *n* = 62, male 35 (56.5%). LOS: 71 (27) days in the CL group; 75 (36) days in DL group.
6. Esmaeilizadeh et al. (2016) / Iran	<35	CL: 33 (2) weeks; CBL: 31 (2) weeks	CL: 1,460 (345) g; CBL: 1,440 (340) g	CL: Open the windows and let in natural light during the day, make up for the deficiency by 200-300 lux; 3-7 lux at night CBL: Open the windows for natural light throughout the day, and make up for 200-300 lux	CL group *n* = 30; CBL group *n* = 30 LOS: 9.1 (1.4) days in the CL group; 10.6 (1.5) days in CBL group Weight gain after birth: CL group 263 (52) g; CBL group 232 (48) g
7. Farahani et al. (2018) / Iran	<35	CL: 32.97 (1.81) weeks; CBL: 32.67 (1.96) weeks	CL: 1,903.0 (340.4) g; CBL: 1,911.0 (363.6) g	CL: NICU 07:00-19:00 Normal NICU light (no clear value); reduced light (use a piece of acrylic glass covered with a dimmable cotton cover to 25 lux from 19:00-07:00) CBL: Continuous light exposure to the research environment throughout the day	CL group *n* = 33, male 25 (75.8%); CBL group *n* = 33, male 17 (51.5%). LOS: CL group 18.39 (12.01) days; CBL group 18.18 (10.21) days Weight gain after birth weight (daily): CL group 29.17 (7.32) g/day; CBL group 14.63 (5.64) g/day
8. Miller et al. (1995) / USA	<31	CL: 28.0 (2.2) weeks; CBL: 28.0 (2.1) weeks	CL: 1,151 (360) g; CBL: 1,049 (330) g	CL:156-201 lux during 07:00-18:00;20-32 lux during 18:00-7:00 CBL: 176-232 lux during the day, 206-274 lux at night	CL group *n* = 20; CBL group *n* = 21. LOS: CL group 59 (27.7) days; CBL group 75 (25.3) days
9. Sánchez-Sánchez et al. (2022) / Mexico	<33	CL: 32.4 (0.2) weeks; CBL: 32.5 (0.1) weeks	CL: 1,655.9 (26.2) g; CBL: 1,629.79 (31.2) g	CL: 07:00 to 19:00 regular room light; 25 lux during 19:00-07:00 CBL:275.82 (14) lux during the day; 145.28 (14) lux at night	CL group *n* = 150; CBL group *n* = 144. LOS: CL group 23 (0.7) days; CBL group 33.77 (1.3) days
10. Vásquez-Ruiz et al. (2014) / Mexico	<37	CL: 31.67(2.40) weeks; CBL: 31.73 (1.35) weeks	CL: 1,563.63 (569.62) g; CBL: 1,452.42 (442.08) g	CL: 249 (11) lux during the day; 27 (0.8) lux during 19:00-07:00 CBL: 249 (11) lux in the whole day	CL group *n* = 19; CBL group *n* = 19. LOS: CL group 34.37 (13.60) days; CBL group 51.11 (23.06) days.

*Note.* Values in the table are mean (*SD*) values. CBL = continuous bright light; ; CL= day and night cycled light; DL = dim light; LOS = length of hospital stay; NICU = neonatal intensive care unit; PMA = postmenstrual age.

### Risk of Bias

**Figure 2:**
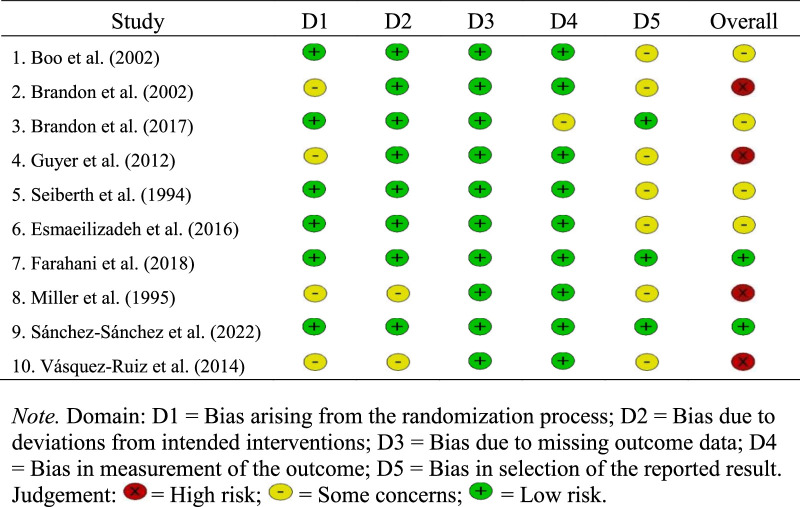
Risk of Bias Summary

Detailed information on the quality, measured in terms of risk of bias, of the 10 included studies is presented in Figure 2. Of these studies, nine focused on LOS and five on weight gain. The interventions were delivered in the NICU. The results of the risk-of-bias assessments were risks of bias due to the randomization process (*some concerns* for 4 studies), deviations from intended interventions (*some concerns* for 2 studies), missing outcome data (*low risk* for all 10 studies), outcome measurements (*some concerns* for 1 study), and results selection (*some concerns* for 7 studies). On the basis of the total risk-of-bias score, two, four, and four studies were deemed to have, respectively, a low risk of bias, some bias concerns, and a high risk of bias.

### Effects of CL Exposure on LOS and Weight Gain

#### Comparison of LOS

**Figure 3:**
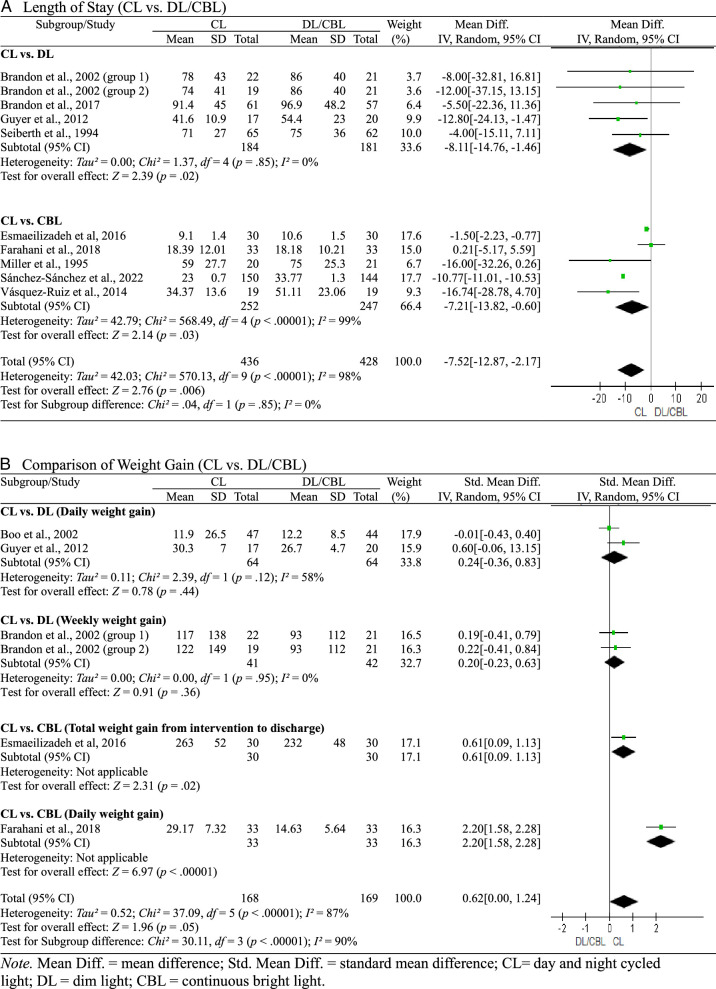
Forest Plot of Length of Stay and Weight Gain

LOS and weight gain data from the 10 studies are depicted in a forest plot in Figure 3. Half of the studies (Boo et al., 2002; Brandon et al., 2002; Brandon et al., 2017; Guyer et al., 2012; Seiberth et al., 1994) compared CL and DL exposure, and the other half (Esmaeilizadeh et al., 2016; Farahani et al., 2018; Miller et al., 1995; Sánchez-Sánchez et al., 2022; Vásquez-Ruiz et al., 2014) compared CL and CBL exposure.

The results of the between-group comparisons for LOS are presented in Figure 3A. In the CL group, LOS was 8.11 days (95% CI [−14.76, −1.46]; *p*=.02) and 7.21 days (95% CI [–13.82, −0.60]; *p*=.03) less than in the DL and CBL groups, respectively. Overall, LOS was significantly lower in the CL group compared with both the DL and CBL groups (MD=7.52 days; 95% CI [−12.87, −2.17]; *p*=.006). Moreover, the level of heterogeneity was high (*I* ^2^=98%, *p*<.00001), and Egger’s test results were not significant (*p*=.23).

#### Comparison of Weight Gain

To compare CL exposure with DL exposure, the cohort was divided into two groups based on the category of weight gain after birth, that is, daily weight gain (Boo et al., 2002; Guyer et al., 2012) and weekly weight gain (Brandon et al., 2002). Because of differences in the unit of time used to calculate weight gain, SMD was used for this comparison. To compare CL exposure with CBL exposure, the cohort was divided into two groups based on the category of weight gain between intervention and hospital discharge, that is, total weight gain and daily weight gain. SMD was calculated to account for the differences in units of time used. The between-group comparison results for weight gain are presented in Figure 3B, with a *p* value of .05 (SMD=0.62; 95% CI [0.00, 1.24]) used as the cutoff threshold for significance. As with LOS, the level of heterogeneity was high (*I* ^*2*^=87%, *p*<.00001), and Egger’s test results were not significant (*p*=.16).

## Discussion

### Principal Findings and Comparisons

LOS values in premature infants between the CL and DL/CBL groups (Brandon et al., 2002; Brandon et al., 2017; Guyer et al., 2012; Seiberth et al., 1994) were compared in this meta-analysis. LOS in the CL group was found to be significantly (8.11 and 7.21 days) less than in the DL and CBL groups, respectively, supporting the potential of CL exposure in reducing LOS in preterm infants. The longer LOS in the DL and CBL groups may raise burdens on both the newborn’s family and the health care system (Farahani et al., 2018). These findings on LOS are similar to those found in another meta-analysis by Morag and Ohlsson (2016), which reviewed only four studies on LOS compared with the nine in this study. Overall, LOS totaled an average 7.52 days less in the CL group than in the DL/CBL group.

In three of the included studies, although weight gain was higher in the CL group than the DL group, this difference did not reach significance (Boo et al., 2002; Brandon et al., 2017; Guyer et al., 2012). Likewise, no significant difference in weight gain was noted between the CL and CBL groups. Similar to this study, Morag and Ohlsson (2016) observed no significant difference between CL and DL exposure in their two included studies on weight gain. In this meta-analysis, a comparison of weight gain among the CL, DL, and CBL groups revealed a *p* value of .05, indicating a need for additional research. However, Esmaeilizadeh et al. (2016) found a significant difference in weight gain between the CL and DL groups (263±52 vs. 232±48 g, respectively; *p*=.02). Moreover, Farahani et al. (2018) reported significant differences between the CL and CBL groups (29.17±7.32 vs. 14.63±5.64 g/day, respectively; *p*<.000001) in daily weight gain from the ninth day after birth until hospital discharge. Although three of the included studies indicated significant weight gain in the CL group compared with that in the CBL group, no data were available for analysis in this study. Sánchez-Sánchez et al. (2022) observed significant differences in weight gain between CL and CBL groups (F(1.897)=100.60; *p*<.0001); Miller et al. (1995) reported the average weekly weight gain to be significantly higher in the CL group than in the CBL group (9.4% vs.7.4%; *p*<.05); and Vásquez-Ruiz et al. (2014) indicated significant differences in weight gain 7 days after birth between the CL and CBL groups (*p*<.001).

In two of the included studies, the CL group exhibited significant weight gains and elevated melatonin levels. In one of these, the CL group exhibited reduced daytime and elevated nighttime salivary melatonin levels as early as day 5 after birth (*p*<.0001; Vásquez-Ruiz et al., 2014), while in the other, the CL group exhibited significant differences (*p*<.0001) between mean daytime and nighttime melatonin levels from the sixth day after birth (Sánchez-Sánchez et al., 2022).

In the late stages of pregnancy, fetal circadian rhythm is regulated by maternal circadian rhythm and, after birth, it is regulated by the artificial environment outside of the womb (Felten et al., 2023). In light of this, the NICU should avoid chaotic, non-circadian-rhythm methods of caring for premature infants. A study involving premature infants revealed that CL exposure improved autonomic circadian rhythms and increased melatonin levels, thereby facilitating the production of growth hormones to promote weight gain and growth (Kennaway et al., 1996). Furthermore, weight gain is influenced by factors such as sleep quality, physiological characteristics (crying, exercise activities, and heart rate), and ventilation duration (days). Disrupted sleep in premature infants may hinder growth and development, leading to delays in hospital discharge (Park, 2020).

Bobinski et al. (2024) reviewed three level I randomized controlled trials regarding the potential of CL exposure to improve sleep in premature infants. Guyer et al. (2012) found that infants exposed to CL cried and exhibited fussiness for shorter periods than those exposed to DL, indicating relatively fast behavioral state regulation in CL-exposed infants. Miller et al. (1995) observed that premature infants exposed to CL exhibited improved motor coordination, a vital factor influencing general development in this population (Fan et al., 2021). Also, Miller et al. reported that infants exposed to CL spent fewer days on ventilators, started oral feeding sooner, and had a higher caloric intake than those not exposed to CL. Brandon et al. (2017) demonstrated hospitalization duration to be associated positively with ventilator usage. Vásquez-Ruiz et al. (2014) found that, compared with preterm infants exposed to CBL, those exposed to CL had a more stable mean heart rate from the third day after birth. The general discharge standard for premature infants includes ensuring appropriate weight gain (Anderson & Narvey, 2022). Therefore, the more weight gained due to CL exposure, the better an infant’s physical function and the easier it is to meet discharge standards, which can shorten LOS for premature infants and reduce medical costs for their families.

### Limitations

This study was influenced by several limitations. First, the small number of included studies advises caution when interpreting the data. Second, the risk-of-bias assessment for eight studies revealed some concerns and high risks that potentially limit the internal validity of the findings. Third, in their two studies conducted in 2002 and 2017, Brandon and colleagues divided premature infants into three and two groups, respectively, comparing LOS and weight gain between CL and DL groups. Their inclusion of infants exposed to CL at 36 weeks PMA in the DL group may have reduced the observed effect of CL exposure. These limitations may compromise the overall credibility of the findings of this meta-analysis.

### Implications for Clinical Practice

The findings of this meta-analysis have several implications for clinical practice. CL was shown to be significantly more effective than either DL or CBL in reducing LOS. Also, despite the limited amount of complete data on weight gain available for analysis, multiple studies have demonstrated CL to be more effective than either DL or CBL in promoting weight gain. Overall, the findings support providing CL exposure to premature infants during their transition period between birth and hospital discharge.

### Implications for Future Research

Given the significant potential benefits of CL and the small sample sizes available for this meta-analysis, large-scale studies are needed to verify the benefits of CL exposure preliminarily identified in the NICU. Furthermore, as light intensity varied across the included studies, instruments should be used in future studies to measure/control for light intensity, reducing the potential for human error. Notably, data should be compared for the period from intervention to hospital discharge. Baseline data should be collected at the initiation of intervention, rather than birth. Because all infants typically lose 10%–15% of their birth weight during the first week after birth before they start to gain weight (Vásquez-Ruiz et al., 2014), overly short LOS durations or overly delayed interventions may allow initial weight losses to affect comparisons of weight gain between the intervention and control groups. Thus, weight gains should ideally be compared only after infants have regained their birth weight. A study conducted in China (2015–2018) reported that weight gains in infants at 24–36, 37–42, and 43–50 weeks PMA were 17–18±2, 10–11±2, and 6 to 7±1 g/kg/day, respectively (Zong et al., 2022). Future studies should indicate both the actual weight and weight gain to facilitate meaningful comparisons of weekly standard weight gains between preterm and term infants. The findings of this meta-analysis revealed that the infants exposed to CL from 32 weeks PMA exhibited greater weight gain (122±149g) than those exposed to CL from birth (117±138 g) or 36 weeks PMA (93±112 g; Brandon et al., 2002). Retinopathy of prematurity affects premature infants born before 31 weeks of gestation (Wilkinson et al., 2023). Therefore, to promote weight gain, CL exposure may be implemented either at 32 weeks PMA or after infants have regained their birth weight.

### Conclusions

The findings of this meta-analysis reveal LOS to be significantly shorter in infants exposed to CL than in those exposed to either DL or CBL, and that CL has a more positive influence on weight gain as well. Overall, CL exposure in preterm infants should be implemented during the transition from NICU to discharge. Future reviews should include studies with sufficiently large sample sizes to confirm the benefits of CL exposure in the NICU. In addition, to avoid retinopathy of prematurity, premature infants should be exposed to CL at 32 weeks PMA or after they have regained their birth weight, with this intervention continued until the time of hospital discharge. Finally, based on the findings, weight gain in preterm infants should be compared against standard weight gain in term infants in future studies as well.
